# Population-based phase II trial of stereotactic ablative radiotherapy (SABR) for up to 5 oligometastases: SABR-5

**DOI:** 10.1186/s12885-018-4859-7

**Published:** 2018-10-04

**Authors:** Robert Olson, Mitchell Liu, Alanah Bergman, Sonya Lam, Fred Hsu, Benjamin Mou, Tanya Berrang, Ante Mestrovic, Nick Chng, Derek Hyde, Quinn Matthews, Chad Lund, Daniel Glick, Howard Pai, Parminder Basran, Hannah Carolan, Boris Valev, Shilo Lefresene, Scott Tyldesley, Devin Schellenberg

**Affiliations:** 1 University of British Columbia, Vancouver, Canada; 2 Unviersity of Northern British Columbia, Prince George, Canada; 3 BC Cancer – Prince George, 1215 Lethbridge Street, Prince George, BC V2M7A9 Canada; 4 BC Cancer – Vancouver, Vancouver, Canada; 5 BC Cancer – Abbotsford, Abbotsford, Canada; 6 BC Cancer – Kelowna, Kelowna, Canada; 7 BC Cancer – Victoria, Victoria, Canada; 8 BC Cancer – Surrey, Surrey, Canada

**Keywords:** Stereotactic ablative radiotherapy, Oligometastases, Radiotherapy

## Abstract

**Background:**

Oligometastases refer to a state of disease where cancer has spread beyond the primary site, but is not yet widely metastatic, often defined as 1–3 or 1–5 metastases in number. Stereotactic ablative radiotherapy (SABR) is an emerging radiotherapy technique to treat oligometastases that require further prospective population-based toxicity estimates.

**Methods:**

This is a non-randomized phase II trial where all participants will receive experimental SABR treatment to all sites of newly diagnosed or progressing oligometastatic disease. We will accrue 200 patients to assess toxicity associated with this experimental treatment. The study was powered to give a 95% confidence on the risk of late grade 4 toxicity, anticipating a < 5% rate of grade 4 toxicity.

**Discussion:**

SABR treatment of oligometastases is occurring off-trial at a high rate, without sufficient evidence of its efficacy or toxicity. This trial will provide necessary toxicity data in a population-based cohort, using standardized doses and organ at risk constraints, while we await data on efficacy from randomized phase III trials.

**Trial Registration:**

Registered through clinicaltrials.gov NCT02933242 on October 14, 2016 prospectively before patient accrual.

**Electronic supplementary material:**

The online version of this article (10.1186/s12885-018-4859-7) contains supplementary material, which is available to authorized users.

## Background

Oligometastases refer to a state of disease where cancer has spread beyond the primary site, but is not yet widely metastatic, often defined as 1–3 or 1–5 metastases in number [1]. It has been proposed that aggressive treatment of oligometastases may improve patient outcomes, but only recently have techniques become widely available to do so non-invasively [2, 3]. Ablation of metastatic deposits can be achieved through several techniques including surgery, radiofrequency ablation, or high-dose radiotherapy [4, 5]. Stereotactic ablative body radiotherapy (SABR), is an emerging radiotherapy technique that delivers very large, hypofractionated doses of highly-conformal radiotherapy to tumor targets with the aid of on-board imaging for accuracy, giving higher rates of local control [6, 7].

Clinical evidence to support the presence of an oligometastatic state is controversial, with low quality evidence in both the surgical and SABR literature [3, 8, 9]. Sufficient level of evidence to support aggressive treatment of oligometastases is lacking, and often based on single-arm studies without appropriate controls [10]. The long-term survival achieved with treatment of oligometastases may be a result of selection bias rather than the result of treatment intervention. There is sparse data on the subacute or long-term effects of SABR treatment. The use of SABR for newly progressive sites (oligoprogression) is also an area of increased interest that has the potential for more widespread use of SABR, further supporting the argument that more robust data on SABR efficacy and toxicity is needed [11].

Given the lack of robust evidence supporting the use of SABR for oligometastases, BC Cancer (British Columbia, Canada) has decided it will not be offered outside of a clinical trial. *The main focus of this trial is to assess the side effects and quality of life post-SABR on a population scale in BC, and to attain more precise estimates of the risk of late effects in long term survivors.* This trial provides a clear informed consent process, including the limited evidence for SABR off trial, and potential harm from SABR, for patients opting to pursue SABR for oligometastases or oligoprogression.

## Methods/design

This is a non-randomized phase II trial where all participants will receive experimental SABR treatment to all sites of progressing metastatic disease. We will accrue 200 patients to assess toxicity associated with this experimental treatment using standardize organ at risk (OAR) constraints (see Additional file 1). The study was powered to give a 95% confidence on the risk of late grade 4 toxicity, anticipating a < 5% rate of grade 4 toxicity. All participants will be accrued with BC Cancer, at each of the 6 BC Cancer regional centres. This study has been approved by the joint UBC/BC Cancer ethics board. All data will remain confidential and stored within the BC Cancer network. Consent will be obtained by a qualified investigator, who will also be a radiation oncologist who prescribes SABR clinically at BC Cancer. Written consent will be obtained.

### Objectives

To assess quality of life and side effects in patients with up to 5 newly diagnosed or progressing metastatic cancer lesions treated with a comprehensive oligometastatic SABR treatment program. Secondarily we will document the disease free and overall survival.

### Primary endpoints

- Patient Reported Outcomes, including quality of life and side effects
- Toxicity Assessed by the National Cancer Institute Common Toxicity Criteria (NCI-CTC) version 4 for each organ treated

### Secondary endpoints

- Progression-free survival
- Time from SABR treatment to disease progression at any site or death
- Overall survival
- Lesion control rate, defined as lack of further progression at the treated site
- Time to starting or re-starting chemotherapy
- Number of cycles of further chemotherapy/systemic therapy

#### Inclusion criteria

- Age 18 or older
- Able to provide informed consent
- Histologically confirmed malignancy with metastatic disease detected on imaging.
  - Biopsy of metastasis is preferred, but not required.
- Primary tumour treated radically or controlled by prior palliative radiotherapy or systemic therapy
- Maximum 5 metastases eligible for SABR (either 5 in total or 5 not controlled by prior treatment)
- Standard of care tests prior to SABR CT simulation within 12 weeks:
  - Brain CT or MRI imaging (for tumor sites with propensity for brain metastasis)
  - Body imaging:
    - CT chest/abdomen/pelvis with bone scan required if no PET-CT is performed
    - PET-CT is only required for specific evidence-based indications, and in such cases the CT neck/chest/abdomen/pelvis and bone scan are not required:
    - MRI spine for patients with vertebral or paraspinal metastases
      - For other indications, at the discretion of the treating oncologists, PET-CT scans may be done but are not required.
  - Liver function tests (AST, ALT, GGT, alkaline phosphatase) for patients with liver metastases
  - Tumor marker testing as appropriate (e.g. CEA for colorectal cancer, PSA for prostate cancer, etc)
  - Pregnancy test for women of child-bearing age
- ECOG performance status 0–2
- All sites of progressive disease can be safely treated based on criteria below
- For non-brainstem mets, maximum size of 3 cm if using single fraction radiosurgery.
  - If size is from 3.1 to 4 cm, 25-35Gy/5 can be considered
- All brain mets cases need approval from Stereotactic Radiosurgery rounds
  - Maximum size of 6 cm for lesions outside the brain, except:
    - Bone metastases over 5 cm may be included, if in the opinion of the local PI it can be treated safely (e.g. rib, scapula, pelvis)
- Life expectancy > 6 months
  - In many scenarios this is best estimated by a multidisciplinary opinion from disease site experts, preferably obtainted at multidisciplinary tumours rounds.
  - The potential treating SABR radiation oncologist reserves the right to require a multidisciplinary note documenting life expectancy, other treatment options and suitability for SABR.
- Not a candidate for surgical resection at all sites: surgery to all sites not recommended by multidisciplinary team, or unfit or declining surgery.
- Not a candidate for an open randomized clinical trial comparing SABR and a standard treatment.
- Prior chemotherapy allowed but no chemotherapy (cytotoxic, immunotherapy or molecularly targeted agents) 48 h prior to first fraction of radiotherapy, during radiotherapy, or for 48 h after last fraction. Certain chemotherapy agents may require a longer break prior to or after SABR if protocols dictate. Hormonal therapy during SABR is allowed.
- Patients with metastases that have been previously treated may be eligible for this SABR protocol:
  - If the previous treatment was systemic therapy, the patient may be eligible, if the metastases have demonstrated a complete radiologic response
  - If the previous treatment was by a local non-radiation means (e.g. prior resection, RFA or microwave ablation), then SABR may be considered for residual/recurrent disease
  - If the previous treatment was SABR, the *patient is not eligible* unless the new site(s) was/were not previously treated
  - If the previous treatment was conventional RT, SABR could be considered if it can be delivered safely. In such a circumstance it *must* be presented in a multidisciplinary setting for approval.

#### Exclusion criteria

- Serious medical co-morbidities precluding radiotherapy
- Bone metastasis in a femoral bone if risk of pending fracture is high
- Patients with 1–3 brain metastasis and no disease elsewhere (these patients should not be accrued but treated with stereotactic radiosurgery or radiotherapy as per results of published randomized trials)
- Complete response to first-line chemotherapy (i.e. no measurable target for SABR)
- Persistent malignant pleural effusion
- Inability to treat all sites of progressing disease with ablative intent
- Clinical or radiological evidence of spinal cord compression
- Dominant brain metastasis requiring surgical decompression
- Pregnant or lactating women

#### Evaluation

Staging/Testing within 12 weeks of accrual

- Brain CT or MRI imaging (for tumor sites with propensity for brain metastasis)
- Body imaging:
- CT neck/chest/abdomen/pelvis with bone scan required if no PET-CT is performed
- PET-CT is only required for specific evidence-based indications, and in such cases the CT neck/chest/abdomen/pelvis and bone scan are not required:
- MRI spine for patients with vertebral or paraspinal metastases
- Liver function tests (AST, ALT, GGT, alkaline phosphatase) for patients with liver metastases
- Kidney function tests (Creatinine, eGFR) for adrenal and kidney patients
- Pulmonary function tests for lung metastases
- Tumor marker testing as appropriate (e.g. CEA for colorectal cancer, PSA for prostate cancer, etc)
- Pregnancy test for women of child-bearing age

### Assessment

- ECOG status
- Charleson Comorbidity Index (CCI)
- Patient Reported Outcomes including Quality of Life assessment using BC Cancer’s Prospective Outcomes and Support Initiative [12]
- Provider Reported Toxicity

#### Intervention

All patients accrued will be treated with ablative techniques (e.g. SABR or surgery) to all sites of active metastatic disease.

#### Dose / fractionation / prescription

The Volume of the PTV receiving 100% of the prescription dose is equal to 95%.

The Volume of the PTV receiving 90% of the prescription dose is greater than 99%.

PTV coverage is secondary to OAR)constraints based on clinical judgement. PTV coverage can be decreased in order to meet OAR constraints. If the PTV coverage by 100% is < 50% of the PTV volume, then patients will not be included in the study. Minimum dose to PTV should be > 50% of prescribed dose.

### Immobilization

- Immobilization devices need to be approved by the practicing Radiation Oncologist(s) and SABR physics and dosimetry staff within the particular institution.
- It is left at the discretion of the treating RO/physics to determine which immobilization device is to be used based on their centre/department specific policy.

### Pre-treatment simulation

- 4DCT should be acquired for tumors which are likely to move from respiratory motion, such and lung, liver and adrenal sites.
- If 4DCT is not possible, 3DCT is acceptable
- The CT slices thickness should be no greater than 3 mm, and pixel sizes should be no greater than 1 × 1 mm. For Spine SABR, the CT slice thickness should be no greater than 2 mm (eg. 1.25 mm) Intravenous contrast may be used at the discretion of the treating radiation oncologist.
- Fiducial markers may be used at the discretion of the treating radiation oncologist.
- When available, 4DCT images should be sent to Treatment Planning System:
  - Reconstructed CTs that display entire range of motion (e.g., 10 phases)
  - MIP (Maximum Intensity Projection)
  - MIN (Minimum Intensity: for bony lesions)
  - Average CT if used for planning purposes

### Treatment volumes

For all lesions, the gross tumor volume (GTV) will be defined as the visible tumor on CT and/or MRI imaging. When used for target definition, MRI and/or PET imaging should be registered with the planning CT. For mobile lesions (e.g. Lung and Liver tumours) an internal GTV (IGTV) or internal CTV (ITV) can be created as per site specific BC Cancer clinical protocols. A Clinical Target Volume (CTV) in general will not be used, except in liver and vertebral spine. For vertebral lesions, the CTV will be as per the BC Cancer spine SABR protocol, based on definitions of the Canadian Cancer Trials Group SC.24 protocol. A Planning Target Volume (PTV) margin of 2–5 mm will be added depending on disease site and local immobilization and image guidance practices: no less than a 2 mm margin may be used for spinal stereotactic treatments and brain tumors, and no less than a 5 mm margin for other sites. For radiosurgery platforms, a PTV margin of 0–1 mm is permitted. In situations where imaging or immobilization may be limited, > 5 mm margins may be considered for non-spinal bone metastases.

Organs at risk visible in the planning CT scan will be contoured.

For spinal lesions, a pre-treatment MRI is required to assess the extent of disease and position of the cord. This must be fused with the planning CT scan. A Planning Organ at Risk Volume (PRV) expansion of no less than 2 mm will be added to the spinal cord, and dose constraints for the spinal cord apply to this PRV. For radiosurgery platforms, a PRV margin of 1 mm is permitted for the spinal cord.

### Relevant organs at risk (OAR)

- The relevant OARs are dependent on the location of the target volume and should be outlined from the simulation CT.
- As a general rule, critical structures *within 5 cm of the PTV* should be contoured.
- Please refer to the Additional file 1 for a list of structures that should be considered for different treatment sites.

#### Treatment planning / technique

##### Technique

- 3DCRT, Intensity Modulated radioterhapy (IMRT), volumetric modulated arc therapy (VMAT) or dynamic conformal arcs delivery techniques are allowed.
- Flattening Filter Free (FFF) beams are encouraged
- Gating or Dynamic Tumour Tracking is allowed

##### Dose calculation algorithm

- Type II only (3D scatter correction dose algorthms, such as Eclipse AAA, Acuros, or Collapsed Cone)
- Inhomogeneity Corrections = ON

Dose grid resolution should be approximately equal to the CT slice thickness, and no larger than 3 mm with a higher resolution for Spine SABR.

##### PTV prescription isodose

- The Volume of the PTV receiving 100% of the prescription dose is equal to 95 % “The Volume of the PTV receiving 90% of the prescription dose is greater than 99 %” PTV coverage may be compromised to achieve dose constraints for critical OARs at the discretion of the radiation oncologist, but PTV coverage by 100% dose must be > 50%
- The hotspot should be in the PTV and not in the adjacent normal tissue. The hotspot should generally be less than 150% of the prescription dose. For lung lesions, the hotspot should not exceed 167% of the prescription dose.

### OAR and Normal tissue dose constraints

- Please refer to Additional file 1 for the OAR dose constraints.
- The dose distribution should conform to the PTV as much as possible. As a guideline for a single lung lesion, please refer to Additional file 1 for dose conformality indices. For multiple lung lesions, the specified dose conformality indices might not be achievable.

### Treatment verification / imaging

- Collision Check: Recommended for all plans containing non-coplanar beams
- Imageverification imaging must be acquired before all fractions (e.g. KV, MV, or CBCT).
- It is recommended that CBCT image guidance is used for all treatment fractions), s indicated in the Table 1
- Post treatment fraction imaging should be applied to assess intrafraction motion.
- If treatment time is expected to exceed 45 min, mid-tx position verification should be performed.

**Table 1:** Dose and fractionations by site with [secondary options in square brackets]

Tumor Location	Description		Total Dose (Gy)	Number of fractions	Dose per fraction (Gy)	Frequency
Lung	Tumors 5 cm or less surrounded by lung parenchyma		48 [54]	4 [3]	12 [18]	Every second day
	Within 2 cm of mediastinum or brachial plexus		60	8	7.5	Daily
Bone	Any bone		35 Gy [24]	5 [2]	7 [12]	Daily
Brain	Stereotactic lesions (no whole brain RT)	< 2 cm	24	1	24	Once
		2–3 cm	18	1	18	Once
		3-4 cm	15	1	15	Once
	If whole brain treated, then simultaneous boost to each lesion		35Gy to metastases 20 Gy whole brain (optional)	5	7 Gy to PTV 4 Gy WBRT	Daily
Liver			54 Gy	3	18	Every second day
Adrenal			60 Gy	8	7.5	daily
Lymph Node			40 Gy	5	8	daily

#### Quality assurance

The contours of the GTV, IGTV, ITV, PTV and all relevant OAR will be evaluated and signed off upon review by a second radiation oncologist. Dose volume histogram parameters will be evaluated by the planning dosimetrist(s), physicist(s) and radiation oncologist(s). Institutional quality assurance rounds may also evaluate the radiation plans and delivery of oligometastases SABR. All plans must be independently verified with measurements and/or with quality assurance software used in the verification of SABR plans.

#### Data and safety monitoring committee

There is no independent data and safety monitoring committee (DSMC) for this study. The DMSC will be made up of the study co-investigators. The DSMC will meet twice annually after study initiation to review toxicity outcomes. If any grade 3–5 toxicity is reported, the DSMC will review the case notes to determine if such toxicity is related to treatment. If the DSMC deems that toxicity rates are excessive (> 25% grade 3 toxicity, or > 10% grade 4 or > 3% 5 toxicity), then the DSMC can, at its discretion, recommend cessation of the trial, dose adjustment, or exclusion of certain treatment sites that are deemed as high-risk for complications.

#### Follow-up schedule

See Table 2 for follow-up schedule:

**Table 2:** Evaluation summary

Day	Tests and Procedures
Every 3 months for the first 2 years	Follow-up appointment with study doctor, physical examination by study doctor (or family doctor if appointment is over videolink or phone) and complete questionnaire Blood tests for certain sites (i.e. liver function tests for patients with liver metastases) Assessment of any side effects or adverse events
Months 3, 6, 12, 18, and 24	CT scan(s) and/or other imaging (MRI, PET or bone scan)^a^
Every 6 months for years 2–6	Follow-up appointment with study doctor, physical examination by study doctor (or family doctor if appointment is over videolink or phone) and complete questionnaire. Assessment of any side effects or adverse events

^a^ imaging is optional for prostate cancer patients with PSA < 5

#### Progressive disease

Participants who develop new, untreated metastatic deposits could be considered for SABR at those sites, if such deposits, or progression at previously stable sites, can be treated safely with SABR. If SABR is not possible, then palliative RT can be delivered if indicated.

Participants who have 1st progression at treated sites or develop new, untreated metastatic deposits, or progression at previously stable sites, will undergo re-staging with CTs and bone scans at the discretion of treating oncologist. Follow-up evaluations and questionnaire completions will be based on schedule 13.1. However, it can be changed at study doctor’s discretion. The questionnaires may be done via mail or telephone call.

Patient Reported Outcomes will be collected using the questionnaires chosen from BC Cancer’s Prospective Outcomes and Support Initiative (POSI: H14–00647) based on body site being treated which is part of standard clinical care, and therefore not outlined in detail here for the trial. For body sites not yet supported by POSI, we will using the Functional Assessment of Cancer Therapy (FACT) quality of life questionnaires that correspond to the tumour site(s) being treated.

#### Physician/registered nurse/other reported outcomes

- ECOG performance status
- Outcomes
  - Next local therapy or chemo-/targeted-therapy start date
  - Date of relapse or new metastases
  - Date of death
  - CTCAE version 4.0 toxicity.

#### Measurement of response

- Survival outcomes: Overall survival will be measured as time until death from any cause, and progression-free survival as time to either progression or death, whichever occurs first.
- Lesion control rate will be assessed retrospectively as RECIST criteria has not been validated in the setting of SABR, and is costly to implement in a prospective cohort.

#### Study endpoints and stopping rules

This study with a sample size of 200 patients has limited power to detect an increased incidence of adverse events reactions and complicates (see Tables below). For this reason, the trial’s power is augmented by a Data and Safety Monitoring Committee (DSMC), which is bound by rules that require the suspension/termination of a trial accrual under certain events as outlined below in Tables 3 and 4.

**Table 3:** Probabilities for exact # of grade 4 toxicity events given true rate of grade 4 toxicities =5%, 4%, 3%, 2% for a sample size = 200

# of grade 4 toxicity events out of a sample size = 200	Probability of exact # of grade 4 toxicity events given true rate of grade 4 toxicities = 5%	Probability of exact # of grade 4 toxicity events given true rate of grade 4 toxicities = 4%	Probability of exact # of grade 4 toxicity events given true rate of grade 4 toxicities = 3%	Probability of exact # of grade 4 toxicity events given true rate of grade 4 toxicities = 2%
0	0.004%	0.03%	0.2%	1.8%
1	0.04%	0.2%	1.4%	7.2%
2	0.2%	1%	4.3%	14.6%
3	0.7%	2.7%	8.8%	19.6%
4	1.7%	5.6%	13.4%	19.7%
5	3.6%	9.1%	16.2%	15.8%
6	6.1%	12.3%	16.3%	10.5%

**Table 4:** 95% confidence interval for true rate of toxicities with sample size = 200

# of grade 4 toxicity events	Upper limits of one-sided 95% confidence interval for true rate of grade 4 toxicities (sample size = 200)
0	1.5%
1	2.3%
2	3.1%
3	3.8%
4	4.5%
5	5.2%
6	5.8%

The primary objective of this study is to assess toxicity post SABR for oligometastases. If any grade 5 SAEs definitely, probably, or possibly related to protocol treatment occur, all co-investigators will be made aware within 1 week of reporting to the principal investigator (PI), and a DSMC meeting will be held within 1 month of the event. During this time, any other patients on treatment to the same body site where the grade 5 toxicity was interpreted to originate, (e.g. adrenal metastases) will have their plan reviewed by the PI to determine if that patients’ treatment should be put on hold prior to DSMC meeting.

If 3 or more grade 5 SAEs meet the definition of unanticipated problem (i.e. unexpected, related and involving greater risk occur during this study, the study will be temporarily put on hold in order to organize a DSMC meeting and initiate conversations with the BCCA/UBC REB. Depending on the results of these meetings the study may be permanently closed or significant modifications may be made and re-reviewed by the research ethics board (e.g. remove treatments of adrenal metastases from the study, or reducing the dose, if toxicity was from this body part treatment with SABR).

For the purpose of reporting this study, we will define SABR for oligometatases to be reasonable safe with the following a priori endpoints. We will report the 95% confidence interval (see table above) that the upper confidence is equal to or lower than. In other wordstoxicities (with sample size of 200) we have 95% confidence the toxicity (whatever grade) is < 5% or lower. Likewise, with 6 toxicities, we have 95% confidence the toxicity is less than 6%.

- < 5% grade 5 toxicity
- < 10% grade 4 toxicity
- < 25% grade 3 toxicity

Note: Due to attrition from death caused by progression, we anticipate the number at risk beyond a year for late events will be 100 cases, if 200 are accrued, and therefore we have provided numbers to demonstrate the estimates of our confidence, which is dependent on the number of patients alive at specific time points.

## Discussion

This phase II trial is unique, in that SABR in BC during the study era is only allowed on study protocol, and therefore the analysis will be population-based. This will allow for a more accurate assessment of toxicity assessments, relatively free from selection bias. It is anticipated that this trial, in combination with results from upcoming randomized phase II trials [13, 14], will lead to randomized phase III trials, where efficacy can be properly assessed.

## Additional file

Additional file 1: Dose constraints used for SABR-5 trial. These are based on the AAPM TG 101, SABR-COMET, SC-24 trials as well as most updated references. If any structure is not listed, the constraints may be calculated using the linear quadratic formula from accepted QUANTEC doses, using an alpha-beta ratio of 3 (except neural structure: alpha-beta of 2) for late effects. (DOCX 134 kb)

## Abbreviations

CTV: Clinical target volume
DSMC: Data & safety monitoring committee
GTV: Gross tumor volume
IGTV: Internal gross tumor volume
IMRT: Intensity modulated radiotherapy
ITV: Internal clinical tumor volume
NCIC-CTC: National Cancer Institute Common Toxicity Criteria
OAR: Organs at risk
PTV: Planning tumor volume
SABR: Stereotactic ablative radiotherapy
SAE: Serious adverse events
VMAT: Volumetric modulated arc therapy
