# Supplementary material for: Population-based phase II trial of stereotactic ablative radiotherapy (SABR) for up to 5 oligometastases: SABR-5
Source: BMC Cancer. 2018 Oct 4;18:954. doi: 10.1186/s12885-018-4859-7 (PMC6172706; doi:10.1186/s12885-018-4859-7)
Supplement: Supplementary file 1 — Dose constraints used for SABR-5 trial. These are based on the AAPM TG 101, SABR-COMET, SC-24 trials as well as most updated references. If any structure is not listed, the constraints may be calculated using the linear quadratic formula from accepted QUANTEC doses, using an alpha-beta ratio of 3 (except neural structure: alpha-beta of 2) for late effects. (DOCX 134 kb) [file 12885_2018_4859_MOESM1_ESM.docx]

**Appendix: Dose constraints**

These are based on the AAPM TG 101, SABR-COMET, SC-24 trials as well as most updated references. If any structure is not listed, the constraints may be calculated using the linear quadratic formula from accepted QUANTEC doses, using an alpha-beta ratio of 3 ( except neural structure: alpha-beta of 2 ) for late effects.

| **OAR** | **SABR ORGAN-AT-RISK (OAR) CONSTRAINTS** | | | | | | | | |
| --- | --- | --- | --- | --- | --- | --- | --- | --- | --- |
|  | Dmax to ≤=0.035cc for all OARs.  Exception: Dmax to 0cc (hard max) 🡪 Spinal cord, Spinal cord PRV, Thecal sac/Cauda Equina | | | | | | | | |
|  | **Fractions** | | | | | | | | |
|  | **2** (for spine mets only as per CCTG SC24) | | **3** | **4** | | **5** | | **8** | |
| **Spinal Cord PRV  (2mm on cord)**  **(or Spinal Canal)** | Dmax ≤ 17 Gy^12^ | | Dmax ≤ 20.3 Gy^13^ | Dmax ≤ 23 Gy ^13^ | | Dmax ≤ 25.3 Gy ^13^ | | Dmax ≤ 30.6 Gy^13^ | |
| **Spinal Cord PRV /Thecal Sac (reirradiation)** | See Table 6 below^11^  (NB: 1^st^ row with 0 nBED not applicable, see above for 1^st^ time spine treatment ) | | | | | | | | |
| **Thecal Sac /  Cauda equina** | Dmax ≤ 17 Gy^12^ | Dmax ≤ 24 Gy^1^  V21.9 Gy ≤ 5 cc^1^ | | | NA | | Dmax ≤ 32 Gy^1^  V30 Gy ≤ 5 cc^1^ | | Dmax ≤ 39 Gy^1*^  V36 Gy ≤ 5 cc^1*^ |
| **Sacral plexus** | Dmax ≤ 26 Gy^12^ (+ nerve roots) | Dmax ≤ 24 Gy^1^  V22.5 Gy ≤ 5 cc^1^ | | | NA | | Dmax ≤ 32 Gy^1^  V30 Gy ≤ 5 cc^1^ | | Dmax ≤ 39 Gy^1*^  V36 Gy ≤ 5 cc^1*^ |
| **Brainstem** | NA | (not medulla)  Dmax ≤ 23.1 Gy^1^  V18 Gy ≤ 0.5 cc^1^ | | | NA | | (not medulla)  Dmax ≤ 31 Gy ^1^  V23 Gy ≤ 0.5 cc^1^ | | NA |
| **Optic Pathway** | Dmax≤ 15.8 Gy^17^  D0.2cc≤ 11.5Gy ^17^ | Dmax ≤ 19.5 Gy^17^  D0.2cc ≤ 15.0 Gy^17^ | | | Dmax ≤ 22.5 Gy^17^  D0.2cc ≤ 17.5Gy^17^ | | Dmax ≤ 25 Gy^17^  D0.2cc ≤ 20 Gy^17^ | | NA  NA |
| **Cochlea** | Dmax ≤ 16.5 Gy^18^ | Dmax ≤ 20 Gy^18^ | | | Dmax ≤ 22.5 Gy^18^ | | Dmax ≤ 25.0 Gy^18^ | | NA |
| **Parotids (each)** | Mean ≤ 7 Gy^12^ | NA | | | NA | | NA | | NA |
| **Pharynx** | Dmax ≤ 20Gy^12^ Mean ≤ 9Gy^12^ | NA | | | NA | | NA | | NA |
| **Larynx** | Dmax ≤ 20Gy^12^ Mean ≤ 9Gy^12^ | NA | | | NA | | NA | | NA |
| **PBT and PT  (prox. bronch tree & prox. trachea)** | Dmax ≤ 20 Gy^12^ | Dmax ≤ 30 Gy ^1^ | | | Dmax ≤ 34.8 Gy^3^ | | Dmax ≤ 40 Gy^1^ | | Dmax ≤ 46.3 Gy^3*^ |
| **Lungs-GTV** | NA | >1500cc ≤ 11.6 Gy^1^  V18 Gy ≤ 10%^3,8*^  Mean ≤ 5.5 Gy ^8*^ | | | >1500cc≤ 11.6 Gy^3^  V20 Gy ≤ 10%^3,8^  Mean ≤ 6 Gy ^8^ | | >1500cc ≤12.5 Gy^1^  V22 Gy ≤ 10%^3,8*^  Mean ≤ 6.5 Gy ^8*^ | | > 1500c ≤ 14 Gy^3*^  V26Gy ≤ 10%^3,8*^  Mean ≤ 7 Gy ^8*^ |
| **Each Lung** | V5 Gy ≤ 35%^12^  V10 Gy ≤ 10%^12^  V20 Gy ≤ 3%^12^  Mean ≤ 5 Gy^12^ | NA | | | NA | | NA | | NA |
| **Chest wall and Ribs:** | Dmax ≤ 36.5 Gy^7*^  V25Gy < 30cc^6*^ | Dmax ≤ 44 Gy ^7^  V30 Gy ≤ 30 cc^6^ | | | Dmax ≤ 50 Gy ^7^  V34 Gy ≤ 30 cc^6^ | | Dmax ≤ 55Gy ^7^  V37 Gy ≤ 30cc^6^ | | Dmax ≤ 68 Gy^7^  V45 Gy ≤ 30 cc^6^ |
| **OAR** | **SABR ORGAN-AT-RISK (OAR) CONSTRAINTS** | | | | | | | | |
|  | Dmax to ≤=0.035cc for all OARs.  Exception: Dmax to 0cc (hard max) 🡪 Spinal cord, Spinal cord PRV, Thecal sac/Cauda Equina | | | | | | | | |
|  | **Fractions** | | | | | | | | |
|  | **2  (for spine mets only as per CCTG SC24)** | **3** | | | **4** | | **5** | | **8** |
| **Brachial Plexus** | NA | Dmax ≤ 24 Gy ^1^  V20.4 Gy ≤ 3cc^1^ | | | Dmax ≤ 26 Gy ^4^  V23.6 Gy ≤ 3 cc ^3^ | | Dmax ≤ 30.5 Gy ^1^  V27 Gy ≤ 3 cc^1^ | | Dmax ≤ 35 Gy ^4^ |
| **Heart / Pericardium** | NA | Dmax ≤ 30 Gy^1^  V24 Gy ≤ 15 cc^1^ | | | Dmax ≤ 34 Gy ^3^  V28 Gy ≤ 15 cc ^3^ | | Dmax ≤ 38 Gy^1^  V32 Gy ≤ 15 cc^1^ | | Dmax ≤ 46 Gy ^2^  V39 Gy ≤ 15 cc ^2^ |
| **Great Vessels** | Dmax ≤ 40 Gy^20^ | Dmax ≤ 44 Gy^20^ | | | Dmax ≤ 49.0 Gy^20^ | | Dmax ≤ 51.5 Gy^20^ | | Dmax ≤ 65 Gy ^2^ |
| **Skin** | Dmax < 26 Gy^1*^  V24 Gy < 10 cc^1*^ | Dmax ≤ 33 Gy^1^  V30Gy ≤ 10 cc^1^ | | | Dmax ≤ 36 Gy ^3^  V33.2 Gy ≤ 10 cc ^3^ | | Dmax ≤ 39.5 Gy^1^  V36.5 Gy ≤ 10 cc^1^ | | Dmax ≤ 48 Gy^3*^  V44 Gy ≤ 10 cc^3*^ |
| **Esophagus** | Dmax ≤ 20 Gy^12^ | Dmax ≤ 27.0 Gy^21^ | | | Dmax ≤ 30 Gy ^3, 21^ | | Dmax ≤ 35 Gy^1, 21^ | | Dmax ≤ 40 Gy ^2^ |
| **Stomach** | Dmax ≤ 20 Gy^12^ | Dmax ≤ 22.2 Gy^1^  (Dmax ≤25 Gy  if PTV close by; MRP to specify^14^) | | | Dmax ≤ 27 ^3^ | | Dmax ≤ 32 Gy^1^  (Dmax ≤ 35 Gy  if PTV close by ; MRP to specify^14^ ) | | Dmax ≤ 40 Gy ^2^ |
| **Duodenum** | Dmax ≤ 20 Gy^12^ | Dmax ≤ 22.2 Gy^1^  (Dmax ≤25 Gy  if PTV close by ; MRP to specify^14^) | | | Dmax ≤ 29.0 Gy^1 (EQD2)^ | | Dmax ≤ 32 Gy^1,22^  (Dmax ≤ 35 Gy if PTV close by ; MRP to specify^14^ ) | | Dmax ≤ 39 Gy ^2^ |
| **Small Bowel**  **(Jejunum/ileum)** | Dmax ≤ 20 Gy^12^ | Dmax ≤ 25.2 Gy^1, 23^ | | | Dmax ≤ 28.5 Gy^23^ | | Dmax ≤ 29.0 Gy^23^  (Dmax ≤ 35 Gy^1^ if PTV close by MRP to specify) | | Dmax ≤ 40 Gy ^2^ |
| **Large Bowel**  **(Colon, Rectum)** | Dmax ≤ 20 Gy^12^ | Dmax ≤ 28.2 Gy ^1^ | | | NA | | Dmax ≤ 38 Gy ^1^ | | Dmax ≤ 46 Gy^1^ |
| **Kidneys (each)** | Dmax ≤ 26 Gy^12^  Mean ≤ 6 Gy ^12^ (*each kidney) | NA | | | NA | | NA | | NA |
| **Renal Cortex (Kidneys)**  **(R & L combined)** | See “Kidneys (each)” | > 200 cc ≤ 14.4 Gy^2^ | | | > 200 cc < 16.2Gy^1*^ | | > 200cc ≤ 17.5 Gy^1^ | | > 200 cc ≤ 21 Gy ^2^ |
| **Liver (Liver minus GTV)** | Dmax ≤ 26 Gy^12^  Mean ≤ 8 - 9 Gy^12^ | >700 cc ≤ 17 Gy^1^ | | | NA | | > 700 cc ≤ 21Gy^1^ | | > 700 cc ≤ 22 Gy^2^ |
| **Bladder Wall** | NA | Dmax ≤ 28.2 Gy^1^ | | | NA | | Dmax ≤ 38 Gy^1^ | | NA |
| **Penile Bulb** | NA | Dmax ≤ 42 Gy ^1^  V21.9 Gy ≤ 3 cc^1^ | | | NA | | Dmax ≤ 50 Gy^1^  V30 Gy ≤ 3 cc^1^ | | NA |
| **Femoral Heads (R & L combined)** | NA | V21.9 Gy ≤ 10 cc^1^ | | | NA | | V30 Gy ≤ 10 cc^1^ | | NA |

***Values are EQD2 conversion from stated reference**

^1^ Benedict SH et al., AAPM TG101, Med.Phys 37(9), 2010 (NB: max pt = 0.035cc)
^2^ Palma et al., SABR-COMET Trial, Palma et al, v.1.9, Apr 2015
^3^ RTOG 0915, <http://www.rtog.org/ClinicalTrials/ProtocolTable/StudyDetails.aspx?study=0915>.

^4^ Forquer JA et al., Radiother Onc, 93:408-413, 2009

^5^ Karlsson K et al., IJROBP, 87(3): 590-595, 2013

^6^ Dunlap NE et al., IJROBP 76(3): 796-801, 2010

^7^ Andolino DL et al., IJROBP 80(3): 692-697, 2011

^8^ Baker R, IJROBP, 85(1):190-195, 2013
^9^ Matsuo Yet al., IJROBP, 83(4): e545-549, 2012.
^10^ Bongers EM et al., Radiother Onc, 109: 95-99, 2013 (VU EMC TO 2013)
^11^ Sahgal A et al., IJROBP, 82(1):107-116, 2012
^12^ Sahgal et al., NCIC CTG Protocol #SC.24. Feb 2, 2017

^13^ Sahgal A et al., IJROBP, 85(2):341-7, 2013 ( Table 5; using 5% risk as per Sahgal ; personal communication Feb 2017 )

^14^ BCCA liver protocol 2011

^15^ Velec M, et al, IJROBP (2017), doi: 10.1016/j.ijrobp.2017.01.221
^16^ Grimm, et al, JACMP (2011), 12(2) 267

^17^Hiniker, et al., Sem Radiat Oncol 26(2), 97-104, 2016. DOI: (10.1016/j.semradonc.2015.11.008)

^18^Rashid, et al., Sem Radiati Oncol 26(2) 105-111, 2016. DOI: (10.1016/j.semradonc.2015.11.004)

^19^Kimsey et al., Sem Radiat Oncol 26(2), 129-134, 2016. DOI: (10.1016/j.semradonc.2015.11.003)

^20^Xue et al, Sem Radiat Oncol 26(2), 135-139, 2016. DOI: (10.1016/j.semradonc.2015.11.001)

^21^Nuyttens et al., Sem Radiat Oncol 26(2), 120-128, 2016. DOI: (10.1016/j.semradonc.2015.11.006)
^22^Goldsmith et al., Sem Radiat Oncol 26(2), 149-156, 2016. DOI: (10.1016/j.semradonc.2015.12.002)

^23^ LaCouture et al., Sem Radiat Oncol 26(2), 157-164, 2016. DOI: ([10.1016/j.semradonc.2015.11.009](https://doi.org/10.1016/j.semradonc.2015.11.009))

**Constraints for Dose Spillage and Conformality**

The R100 (ratio of size of prescription isodose volume to size of PTV) should be less than 1.2. Exceptions are allowed for small PTVs. The R50 (ratio of size of 50% prescription isodose volume to size of PTV) should be as low as possible and conform to the table below. Values for the maximum dose 2 cm or more away from the PTV (D2cm) is below.


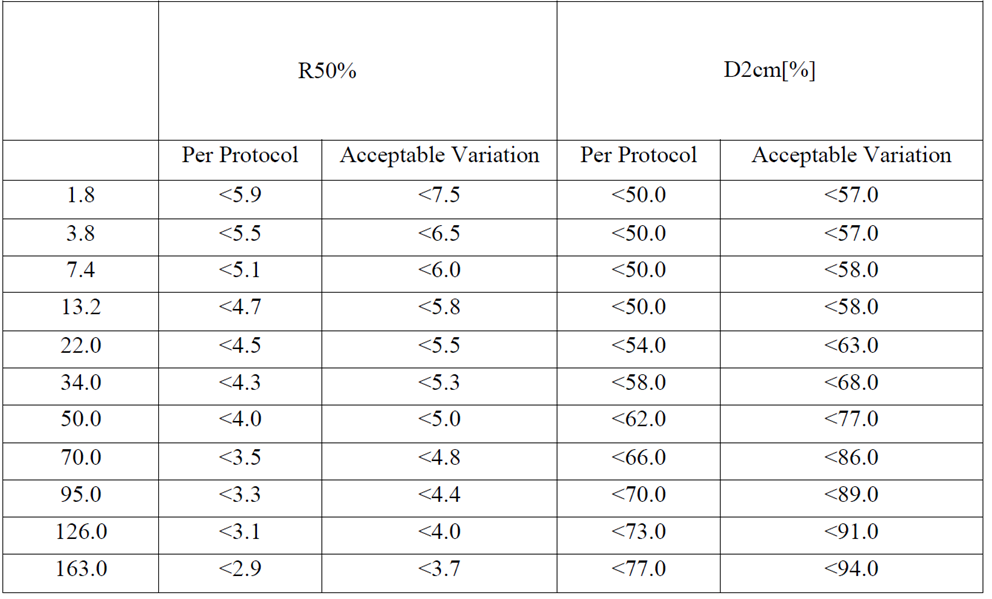


Source: NRG LU002 trial protocol
